# Angioimmunoblastic T-cell Lymphoma: An Unusual Case in an Octogenarian

**DOI:** 10.7759/cureus.6956

**Published:** 2020-02-11

**Authors:** Tejaswi Kanderi, Siddharth Goel, Isha Shrimanker, Vinod K Nookala, Pratiksha Singh

**Affiliations:** 1 Medicine, University of Pittsburgh Medical Center Pinnacle, Harrisburg, USA; 2 Internal Medicine, University of Pittsburgh Medical Center Pinnacle, Harrisburg, USA; 3 Internal Medicine, Hackensack Meridian Health, Ocean Medical Center, Brick, USA

**Keywords:** angioimmunoblastic t-cell lymphoma, lymph node, hepatosplenomegaly, lymphadenopathy

## Abstract

Angioimmunoblastic T-cell lymphoma (AITL) is an unusual subtype of mature peripheral T-cell lymphoma originating from the follicular T-helper cells and is often associated with autoimmune disorders. AITL is an aggressive lymphoma, presenting with constitutional symptoms, generalized lymphadenopathy, and hepatosplenomegaly. Immunohistochemistry and biopsy are diagnostic methods. The treatment modalities range from steroids, immunomodulators, and cytotoxic chemotherapy.

An 87-year-old female presented to the emergency department with cough, dyspnea, dizziness, night sweats, and unintentional weight loss with multiple discrete swellings over her body for a duration of three days. Her physical exam was significant for tachycardia with dry mucous membranes and generalized lymphadenopathy. However, no hepatosplenomegaly was noted. Laboratory investigations revealed neutrophilic leukocytosis (12.8 K/uL), with elevated inflammatory markers (C-reactive protein of 1.39 mg/dL, sedimentation rate of 86 mm/hour). The biopsy of the cervical lymph node revealed atypical lymphoid infiltrates. Flow cytometry showed CD10+ and CD4+/CD8+ T-cells with a minority of CD23+ B-cells, and fluorescence in situ hybridization (FISH) reported gains of the BCL2 gene region on chromosome 18, all of which were suggestive of AITL. She was transferred to an advanced hematology center for staging and targeted therapy.

A careful review of the patient with the prompt clinical and histological examination is essential for the correct diagnosis as the differentials are vast due to its non-specific clinical presentation and accurate treatment is a must for complete remission.

## Introduction

Peripheral T-cell lymphoma is a subtype of non-Hodgkin’s lymphoma. Angioimmunoblastic T-cell lymphoma (AITL) is an unusual subtype of mature peripheral T-cell lymphoma [1]. In the 1970s, it was defined as a separate clinicopathological entity [2]. AITL constitutes approximately 1% to 2% of non-Hodgkin’s lymphoma and about 15% to 20% of peripheral T-cell lymphoma. In the United States, the incidence rate of AITL is only 0.05 new patient cases per 100,000 people [3]. The median age of onset is 60 years or above. It is frequently associated with autoimmune disorders, like immune complexes disorders, autoimmune hemolytic anemia, and rheumatoid arthritis, in about 20% of patients [4-5]. AITL presents with constitutional symptoms, such as fever, significant weight loss, generalized lymphadenopathy, and hepatosplenomegaly [6]. AITL originates from the follicular T-helper cells [1].

Immunohistochemistry is characterized by CD2, CD3, CD4, CD10, CD20, CXCL‐13, PD-1, and BCL6 [7]. Eighty to ninety-five percent of the biopsies involving AITL are positive for Epstein-Barr virus (EBV) [8]. Steroids, immunomodulators, and cytotoxic chemotherapy are the few treatment options that have been utilized to treat AITL [9]. AITL is an aggressive tumor with a poor prognosis. Despite treatment, the average duration of survival is less than three years [5].

## Case presentation

An 87-year-old female presented to the emergency department with worsening lightheadedness, exertional dyspnea, and non-productive cough, along with dizziness, fatigue, night sweats, unintentional weight loss, anorexia, and intermittent nausea. She had three days’ duration of multiple discrete swellings over her body. She had no complaints of fever, hemoptysis, vomiting, blurry vision, palpitations, or calf tenderness. She had a history of coronary artery disease, hyperlipidemia, chronic obstructive pulmonary disease, pulmonary hypertension, gastroesophageal reflux disease, and hypothyroidism. On examination, she was afebrile and tachycardic (108 beats/minute) with a blood pressure of 144/66 mmHg and oxygen saturation of 93% on room air. Further examination was significant for dry mucous membranes, generalized lymphadenopathy, and the absence of hepatosplenomegaly. The rest of the physical exam was unremarkable. Laboratory investigations revealed leukocytosis (12.8 K/uL) with a high neutrophil:lymphocyte ratio (76.8:9.1), hyponatremia (132 mmol/L), hypomagnesemia (1.1 mg/dL), blood urea nitrogen (28 mg/dL), C-reactive protein (1.39 mg/dL), and a sedimentation rate of 86 mm/hour. Urinalysis revealed leukocyte esterase +2, white blood cells 6 - 10/hpf (high power field), and bacteriuria.

Magnetic resonance imaging (MRI) of the neck revealed significant right-sided lymphadenopathy. Echocardiography revealed grade 1 diastolic dysfunction with a preserved ejection fraction. An MRI of the brain was unremarkable. 

Biopsy of the cervical lymph node demonstrated an enlarged lymph node with effaced architecture by atypical lymphoid infiltrate with mixed abundant small lymphocytes, eosinophils, plasma cells, and some histiocytes. The atypical cells had abundant clear to eosinophilic cytoplasm, rough chromatin, round to irregular nuclei, and inconspicuous nucleoli. The immune stains demonstrated increased endothelial cells, focally positive multiple myeloma oncogene 1 (MUM1) cells, and high Ki67 (average 40%) but were largely negative for epithelial membrane antigen (EMA). The antigenic markers that were found to be positive on the immunostaining results are noted in Table 1.

**Table 1:** Immunostaining Results EBER: Epstein-Barr virus‐encoded ribonucleic acid

	Antigenic marker
B-cells	CD20/PAX5, CD138 (scattered), CD15 (scattered and likely eosinophils), EBER
T-cells	CD3/CD5, CD8
Atypical lymphoid cells	CD10, CD30, CD4, PD1, CXCL13, and BCL6

Flow cytometry analysis revealed 96% of total cells as lymphocytes, out of which 59% were B-cells and 38% were T-cells. It also showed a small population of CD10+ T-cells and CD4/CD8 double-positive T-cells. A small population of B-cells that was CD23+ and CD5- was also polyclonal for kappa and lambda light chains. The plasma cells were increased but were polyclonal and showed non-aberrant antigen expression. A polymerase chain reaction (PCR) study for B-cell and T-cell reported positive for T-cell receptor (TCR) beta and gamma gene arrangement; it was negative for B-cell gene arrangement. The fluorescence in situ hybridization (FISH) study reported gains of the BCL2 gene region on chromosome 18.

**Figure 1:**
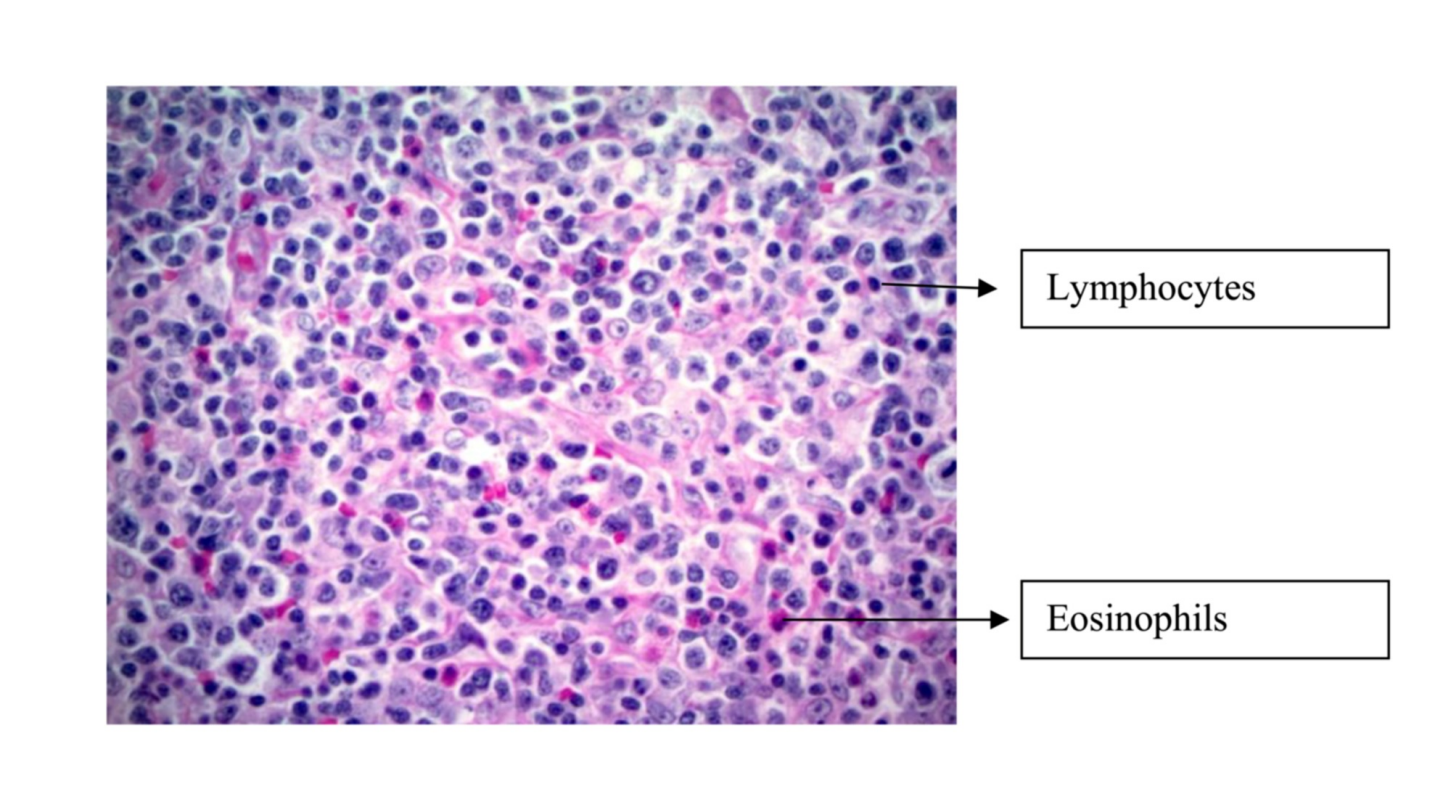
Biopsy specimen in 40x, showing polymorphic infiltrate including eosinophils and small lymphocytes

The biopsy specimen of the right cervical lymph node revealed T-cell lymphoma favoring AITL (Figure 1).

**Figure 2:**
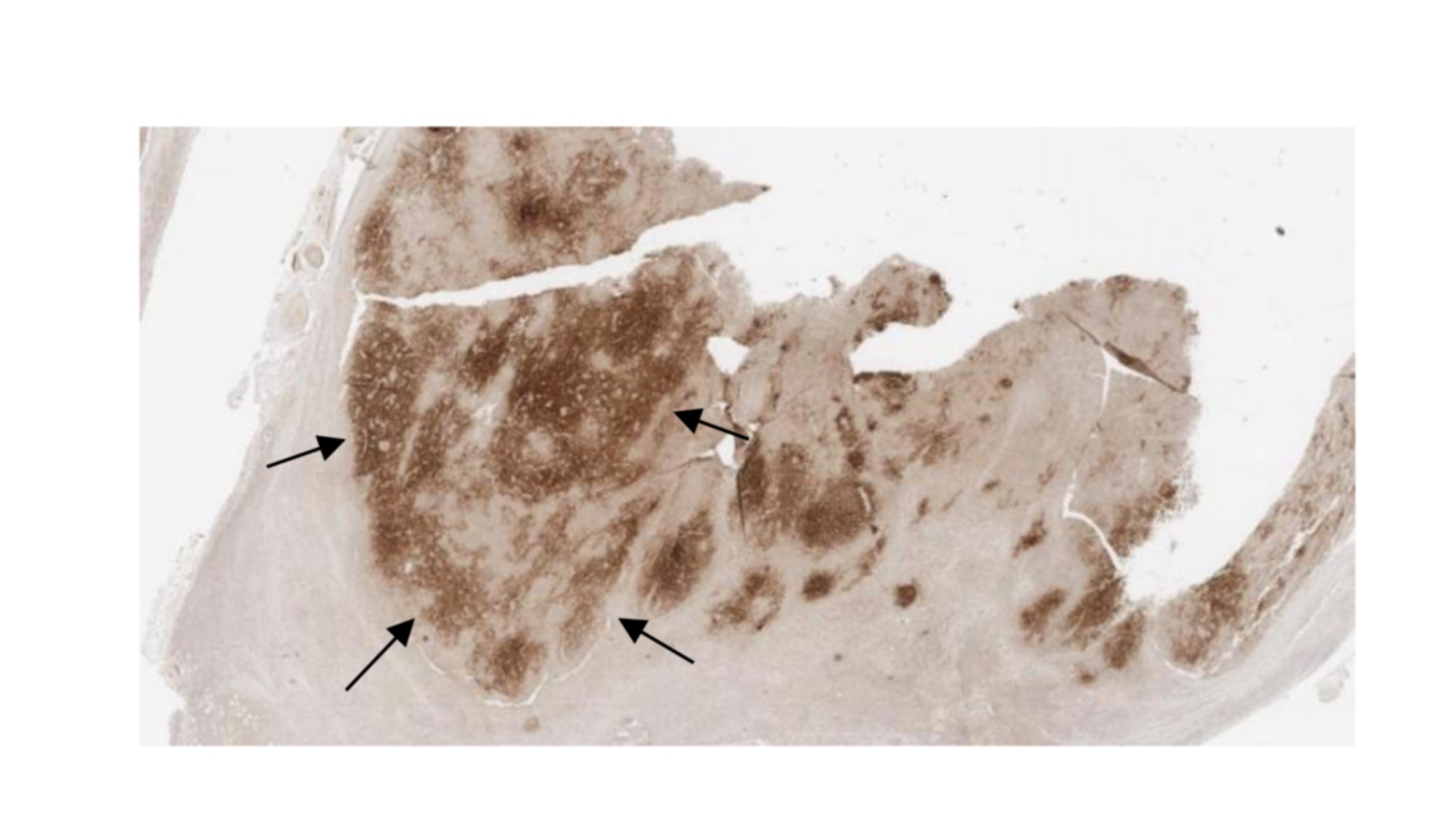
Immunostain positive for CD 21

**Figure 3:**
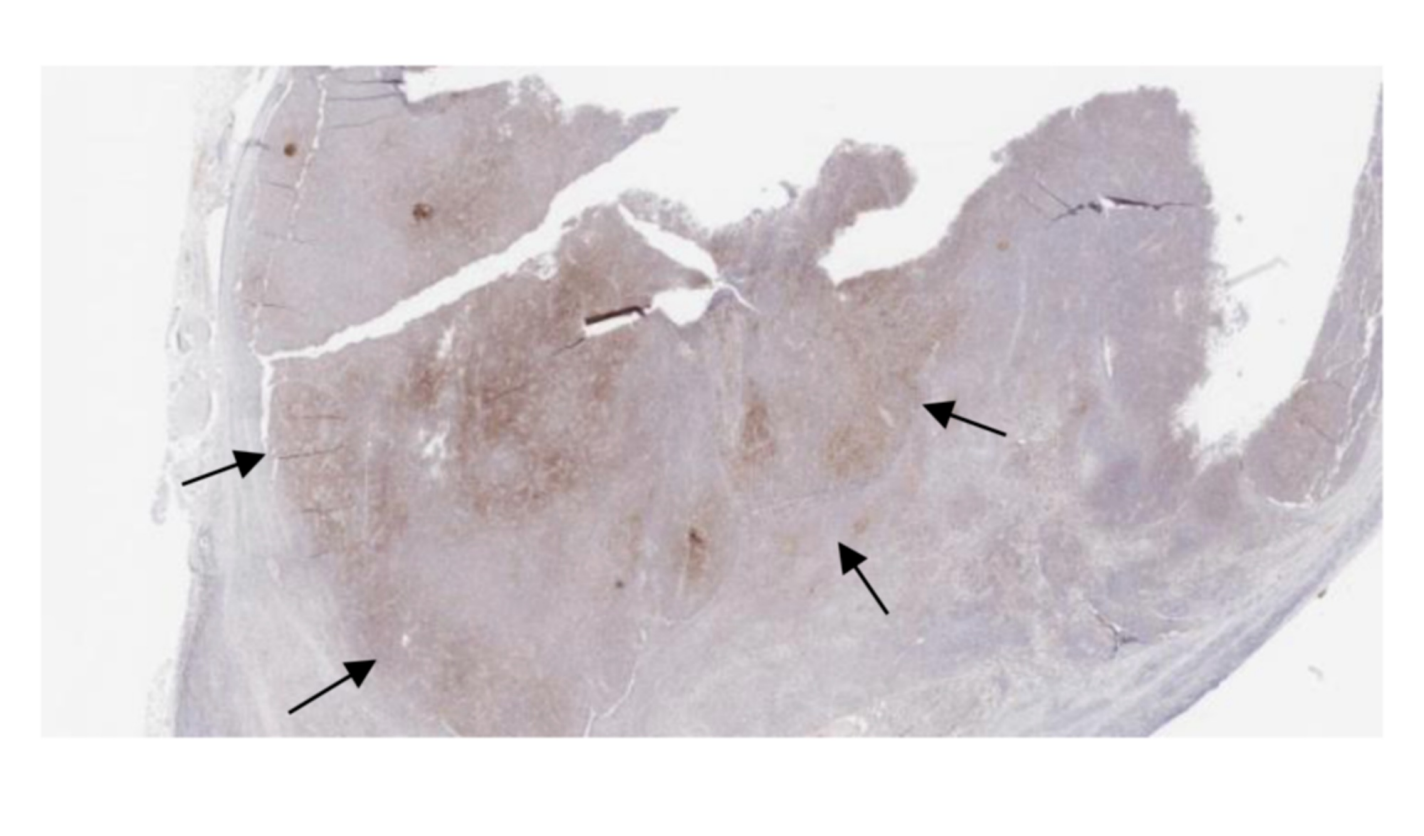
Immunostain positive for CXCL 13

**Figure 4:**
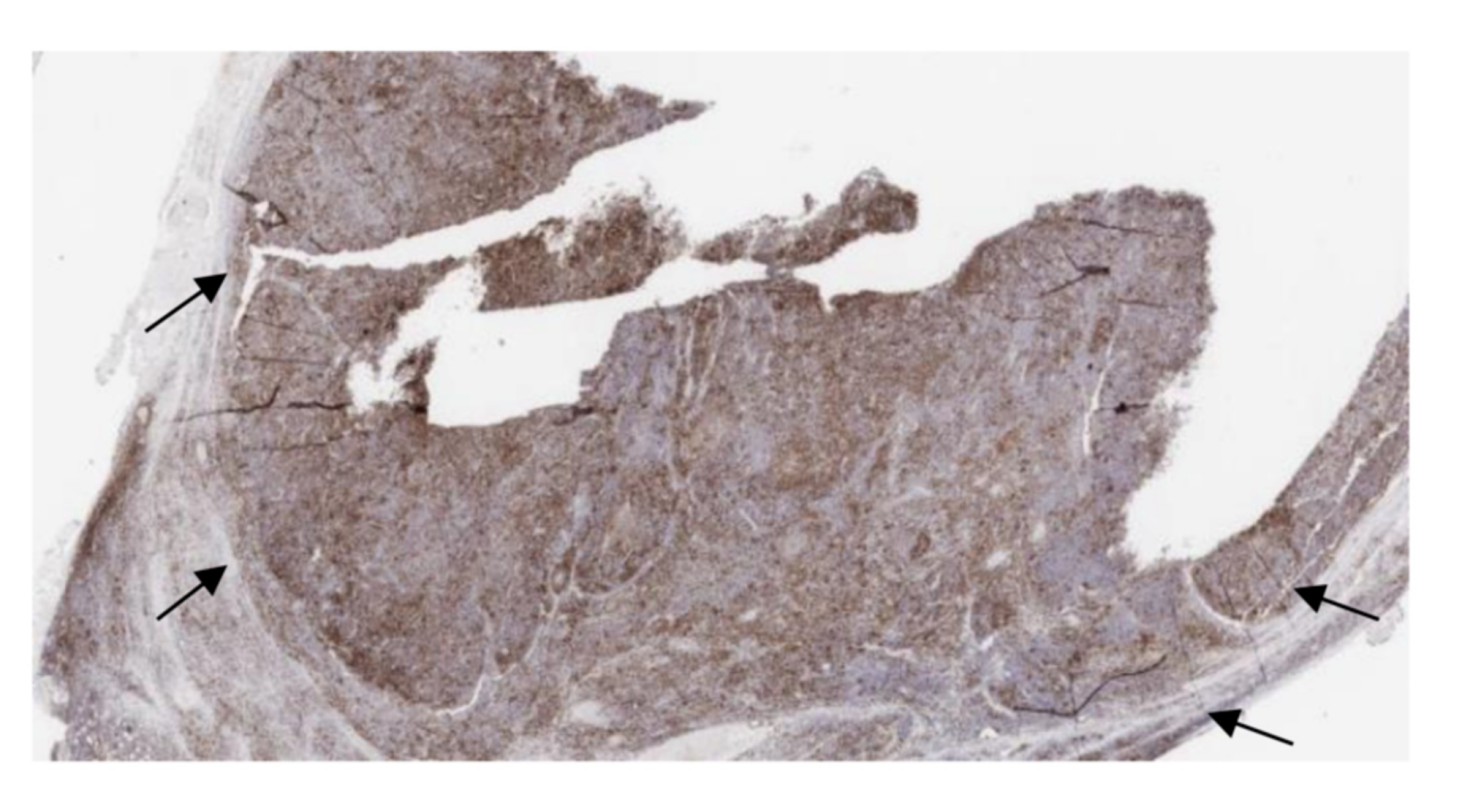
Immunostain positive for PD1

Immunostaining results are revealed in Figures 2-4.

The patient was transferred to an advanced hematology center for staging and targeted therapy.

## Discussion

AITL is an aggressive malignancy with non-specific symptoms that may commonly be present, along with infections and autoimmune disorders. Excisional lymph node biopsy is the gold standard to diagnose AITL. A core biopsy is usually performed to avoid invasive procedures, but they are often insufficient to provide the correct diagnosis.

Most of the patients present with advanced-stage disease (Stages III-IV) at the time of diagnosis [9]. B symptoms are positive in 70% of the patients and 79% of them have splenomegaly. Cutaneous lesions vary widely and can be encountered in approximately half of the cases, manifesting as nonspecific macules, papules, and sometimes purpura, urticaria, nodules, or petechiae. Our case had an atypical presentation, with a later age of presentation and without any presenting symptoms of fever or hepatosplenomegaly. Also, our patient did not have any significant rash. The usual lab findings, such as anemia, lymphocytopenia, and thrombocytopenia, were absent in our patient, whereas neutrophilia was present.

The pathogenesis of AITL is still unclear. It involves clonal rearrangements of T-cell receptor genes. Genomic sequencing shows the presence of acquired "driver" mutations in genes linked with hematologic cancers. The primary site of AITL is a lymph node. The most common presentation is generalized lymphadenopathy. Although most cases of AITL are complicated with the Epstein-Barr virus (EBV) infection, the neoplastic T-cells are frequently EBV-negative. EBV most commonly infects B-cells, and those infected show normal histological findings [4]. In our case, the patient had no history of EBV infection. The histopathology of the lymph nodes showed almost complete effacement of the follicular architecture, a mixed lymphoid infiltrate, and several high endothelial venules in an expanded T-cell zone.

In some cases, the lymph nodes reveal obliteration of the architecture diffusely by infiltration with lymphoid cells, including immunoblasts, lymphocytes, histiocytes, and plasma cells, along with several endothelial venules that are surrounded by a network of follicular dendritic cells. On immunohistochemical staining, the malignant cells express CD2, CD3, and CD5 which are pan T-cell antigens. The predominant proliferating cells are positive for CD4 and sometimes for CD8 as well [3]. A study conducted by Attygalle et al. reported that CD10 distinctly helped in identifying 90% of AITL tumor cells [10]. Dorfman et al. showed that programmed death-1 (PD-1), a component of the CD28 co-stimulatory receptor family is expressed by germinal center-associated T-cells in AITL [11]. De Leval et al. indicated tumor cells in AITL could overexpress CXCL13, which is a characteristic of native follicular T-helper cells [12]. 

This literature suggests that CD10 and PD-1 immunophenotypes are helpful to distinguish AITL from atypical paracortical hyperplasia and other peripheral T-cell lymphomas, as well as for diagnosing extranodal dissemination of AITL. In our patient, not only the histological examination of the lymph node demonstrated a morphologic characteristic of AITL, but also the presence of atypical lymphoid cells with positive CD4, CD10, PD1, CXCL13, and BCL6 expression identified by immunohistochemical staining strongly supported a diagnosis of AITL at the initial lymph node biopsy.

The lack of diagnostic criteria based on clinical and histological features has deemed the diagnosis of AITL challenging [9]. Although AITL may be diagnosed clinically, immunophenotyping is considered an absolute requirement to confirm it [3]. Additional diagnostic markers that might help to improve the chances of diagnosing AITL are CXCL13 and CD279 (also known as PD-1) [5]. The prognostic factors in AITL remain controversial. Pangalis et al. found that the prominence of lymphocytopenia is associated with increased mortality in AITL [13]. Aozasa et al. suggested that clear and convoluted cells on biopsy indicated a poor prognosis [14]. Complete remission after successful treatment is an important prognostic factor. The Kiel Lymphoma Study Group found that survival was significantly related to age, stage, systemic symptoms, skin rash/pruritus, edema, ascites, high lactate dehydrogenase (LDH), and decreased hemoglobin [15]. Archimbaud et al. mentioned prognostic factors associated with shorter survival, including elevated LDH, rash, lymph node eosinophilia, and drug exposure, concerning the onset of the disease; factors associated with prolonged survival were localized lymphadenopathy and successful remission [16]. 

Anthracycline-based therapy is believed to be the first-line treatment among the different regimens used for the treatment of AITL [17]. Complete remission has been attained in 61% of cases treated with this regimen, with a five-year overall survival rate of 32% and recurrence-free survival of 18% [5]. When high-dose chemotherapy, followed by autologous stem cell transplantation, is administered, the likelihood of complete remission is improved.

## Conclusions

In this case report, we describe a case of AITL in an 87-year-old patient who presented with vague and nonspecific symptoms. Although AITL can be diagnosed clinically, histological examination and immunophenotyping of the lymph node upon biopsy confirmed the diagnosis in our patient. A careful review of the patient with the prompt clinical and histological examination was essential for an accurate diagnosis as the differentials are vast and accurate and targeted treatment is a must for complete remission.
